# Dementia in health claims data: The influence of different case definitions on incidence and prevalence estimates

**DOI:** 10.1002/mpr.1947

**Published:** 2022-09-27

**Authors:** Oliver Riedel, Malte Braitmaier, Ingo Langner

**Affiliations:** ^1^ Leibniz Institute for Prevention Research and Epidemiology – BIPS Bremen Germany

**Keywords:** Alzheimer's disease, dementia, epidemiology, health claims data, vascular dementia

## Abstract

**Objectives:**

The epidemiology of dementia subtypes including Alzheimer's disease (AD) and vascular dementia (VD) and their reliance on different case definitions (“algorithms”) in health claims data are still understudied.

**Methods:**

Based on health claims data, prevalence estimates (per 100 persons), incidence rates (IRs, per 100 person‐years), and proportions of AD, VD, and other dementias (oD) were calculated. Five algorithms of increasing strictness considered inpatient/outpatient diagnoses (#1, #2), antidementia drugs (#3) or supportive diagnostics (#4, #5).

**Results:**

Algorithm 1 detected 213,409 cases (#2: 197,400; #3: 48,688; #4: 3033; #5: 3105), a prevalence for any dementia of 3.44 and an IR of 1.39 (AD: 0.80/0.21, VD: 0.79/0.31). The prevalence decreased by algorithms for any dementia (#2: 3.19; #3: 0.75; #4: 0.04; #5: 0.05) as did IRs (#2: 1.13; #3: 0.18; #4: 0.05, #5: 0.05). Algorithms 1–2, and 4–5 revealed similar proportions of AD (23.3%–26.6%), VD (19.9%–23.2%), and oD (53.1%–53.8%), algorithm 3 estimated 45% (AD), 12.1% (VD), and 43.0% (oD).

**Conclusions:**

Health claims data show lower estimates of AD than previously reported, due to markedly lower prevalent/incident proportions of patients with corresponding codes. Using medication in defining dementia potentially improves estimating the proportion of AD while supportive diagnostics were of limited use.

## 1 INTRODUCTION

Dementias, including the subtypes Alzheimer's disease (AD), vascular dementia (VD), and other forms of dementia (oD) are the most common neurodegenerative diseases in older people (Berr et al., 2005), with an estimated prevalence of 12.4% and an estimated incidence of 47.4/1000 person‐years among those aged 75 years and older (Riedel‐Heller et al., 2001a, 2001b). Earlier results from the Rotterdam study suggest that AD accounts for the majority of all dementia cases with an estimated 70%, while approximately 15% suffer from VD (Ott et al., 1995). The epidemiology of dementia can be considered well studied, and in recent years, results from field studies have been increasingly supported by findings based on health claims data (Doblhammer, Fink, & Fritze, 2015; Doblhammer, Fink, Zylla, & Willekens, 2015; Kaduszkiewicz et al., 2014; Stock et al., 2018). Claims data offer a number of research opportunities which can be realized only at very high costs and efforts in field studies, such as the description of multidisciplinary and multisectoral care, including precise documentations of drug treatment patterns in a large number of patients over a long period of time (Gansen, 2018).

However, the majority of studies based on claims data depict the epidemiology of dementia as a whole without differentiating between dementia subtypes. One reason for this might be that many physicians initially code dementia diseases as “unspecified dementia” in the billing process. As it is not necessary to specify the initial diagnosis for billing of ongoing treatment this might lead to a significantly lower prevalence of AD and VD compared to oD observed in these data. Furthermore, these studies are commonly based on algorithms, which either require at least an inpatient diagnosis or two subsequently coded outpatient diagnoses within a specific time. Data are needed on how dementia estimates change if stricter criteria for algorithms are considered, which could corroborate the diagnosis coded in health claims data. These criteria could include, for example, diagnostic procedures explicitly recommended in guidelines for confirming diagnoses (Hofmann et al., 2019; Waldemar et al., 2007).

Therefore, based on a large health claims database, this paper aims at comparing the impact of five different dementia algorithms on the (a) prevalence, (b) incidence, and (c) proportion of AD, VD, and oD among dementia cases.

## 2 METHODS

### 2.1 Data source

This study was based on the German Pharmacoepidemiological Research Database (GePaRD) which comprises claims data from four statutory health insurance providers in Germany and currently includes information on approximately 25 million persons who have been insured with one of the participating providers since 2004 or later (Haug & Schink, 2021). GePaRD comprises demographic information, data on drug dispensations including the anatomical‐therapeutic‐chemical (ATC) code and outpatient and inpatient services, which are coded according to the German Uni‐form Reimbursement Catalog (*Einheitlicher Bewertungsmaßstab,* EBM) for outpatient services and the Operation and Procedure Code (*Operationen‐ und Prozedurenschlüssel,* OPS) for inpatient services, respectively. Diagnoses are coded according to the *International Statistical Classification of Diseases and Related Health Problems 10th Revision, German Modification* (ICD‐10‐GM). In GePaRD, there is information on approximately 20% of the general population available and all geographical regions of Germany are represented.

### 2.2 Study design/study cohorts

The epidemiological estimates were calculated with different analyses based on the calendar years 2004–2016. The prevalence of dementia was estimated annually in cross‐sectional analyses. To be eligible, subjects had to be 50 years of age or older and had to be insured for at least 183 days during the study period. This minimum time frame was chosen because some algorithms (see next section) required the confirmation of a quarterly dementia diagnosis in the following quarter. In each year, prevalence of dementia was assessed for subjects diagnosed with AD (ICD‐10 codes F00 or G30), VD (F01) or oD (F02, F03, F05), according to different algorithms as described below.

**FIGURE 1:**
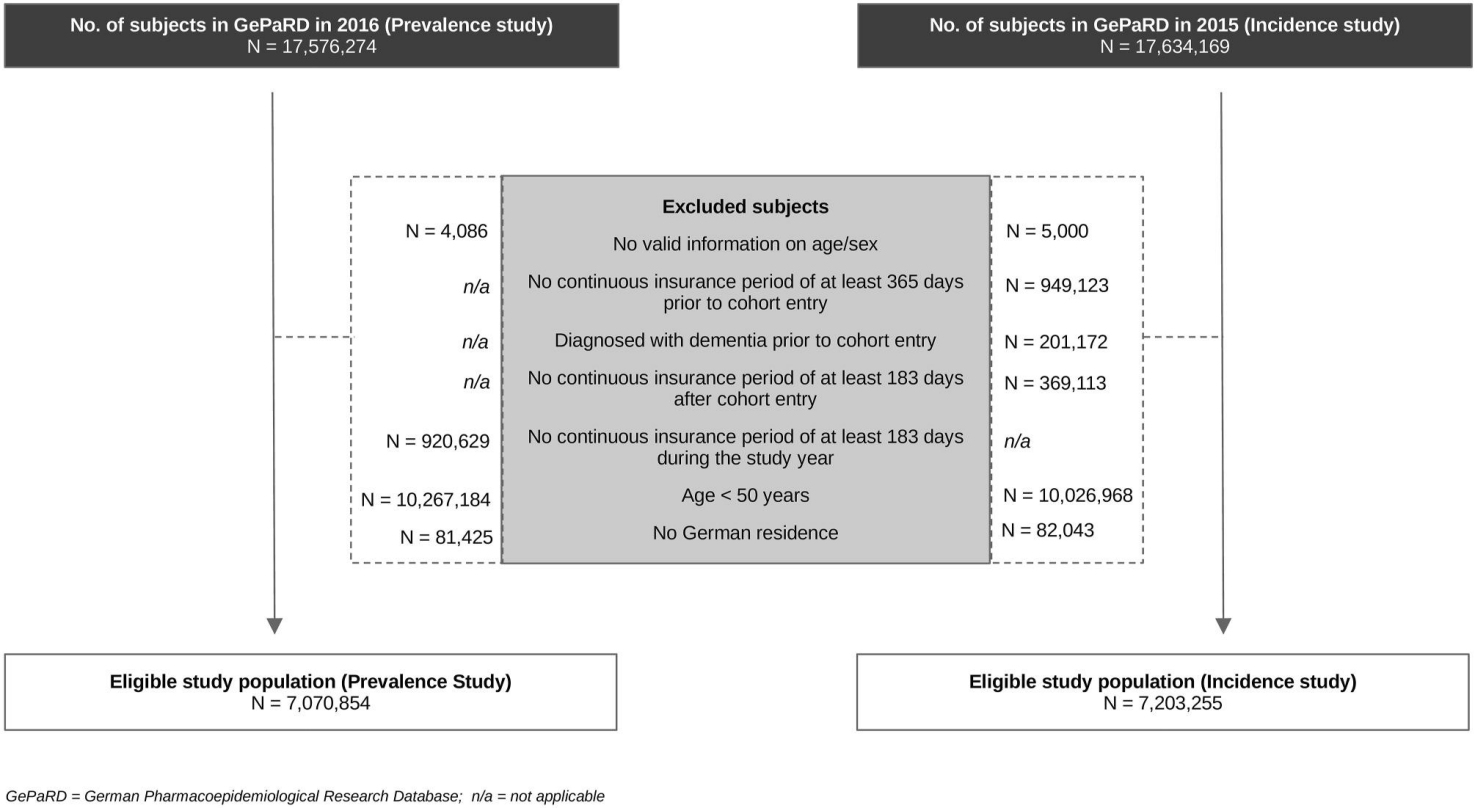
Attrition of the study populations (prevalence and incidence study)

The incidence of dementia was calculated in annual cohorts. To be eligible, subjects had to be 50 years of age or older, had to be insured anytime between 2004 and 2016 and had to be free of any dementia diagnosis or prescription of antidementia drugs for 365 days prior to cohort entry. Cohort entry was defined as the first date after a continuous insurance period of 365 days (thus making January 1st the earliest possible cohort entry in each data year). Cohort exit was defined as (a) the occurrence of AD, VD or oD (see ICD‐10 codes above) or (b) reaching December 31st in the respective data year or 182 days before death or end of insurance period. Persons with more than one dementia diagnosis at cohort exit (e.g., diagnosed with AD and VD on the same day) were assigned to a dementia category according to the following hierarchy: AD > VD > oD. Based on previous epidemiological findings (Ott et al., 1995), this hierarchy was chosen under the assumption that AD is the most common form of dementia, followed by VD and oD. Due to the assessment of the baseline period prior to cohort entry and the use of algorithms for detecting dementia (see next section), which partly required cross‐calendar year analyses, incidence rates (IRs) were not calculated for the years 2004 and 2016. Please note that because of space limitations only data from the last year available will be presented (i.e., prevalence: 2016, incidence: 2015). Figures reflecting earlier data years did not differ substantially from those presented in this paper and are available upon request. Figure 1 shows the attrition of the study populations for the prevalence and incidence analyses. After applying the defined exclusion criteria, the study populations consisted of 7,070,854 potentially eligible subjects for the prevalence study and 7,203,255 potentially eligible subjects for the incidence study (see Figure 1).

The prevalent proportions of dementia subtypes were calculated by dividing the number of patients assigned to a dementia category by the number of all dementia cases (“any dementia”) in the respective year. The incident proportions were calculated likewise in a cohort of patients with incident dementia diagnoses of all years and an available follow‐up of at least 2 years.

### 2.3 Algorithms

For the detection of dementia in the study population, five different algorithms were applied:

Algorithm 1 required at least one inpatient or outpatient diagnosis of dementia. Algorithm 2 required (a) at least one inpatient diagnosis or (b) at least one outpatient diagnosis by a neurologist or (c) one outpatient diagnosis of any specialty and at least another subsequent inpatient or outpatient diagnosis within 183 days. Algorithms 3–5 additionally required that the dementia diagnosis had to have occurred in temporal proximity to another intervention potentially specific for dementia. The underlying methodological rationale for this approach was that a specific dementia diagnosis may be considered more valid if it is associated with such an intervention. Thus, for algorithms 3–5, prescriptions of antidementia drugs and supportive diagnostics (see Supplement Table S1) were considered as follows: Algorithm 3 applied the same criteria as algorithm 2 with the additional requirement of at least one prescription of an antidementia drug within 183 days. Following recommendations for diagnosing dementia (Livingston et al., 2017), algorithm 4 required at least one inpatient or outpatient diagnosis and conduct of laboratory testing (e.g., testing of cerebrospinal fluid or blood for biomarkers) within 183 days. Algorithm 5 required (a) at least one inpatient or outpatient diagnosis and conduct of laboratory testing within 183 days (as in algorithm 4) or (b) one inpatient or outpatient diagnosis and conduct of functional imaging.

### 2.4 Statistics

Prevalence estimates were calculated using a mid‐year population, comprising all individuals with valid information on sex and age with residency in Germany, who were insured on July 1st and the following 182 days of the respective year. The prevalence was then estimated for each algorithm as the number of individuals in an age and sex stratum with a dementia diagnosis (as per algorithm) in a given year, divided by the number of individuals in that age and sex stratum in the mid‐year population. Prevalence is reported per 100 persons. IRs within age and sex strata were calculated as the number of incident dementia diagnoses in the cohort in a given year divided by the person‐time under risk during that year. IRs are reported per 100 person‐years. Both prevalence and incidence estimates were age‐ and sex‐standardized using the European standard population of 2013 (Gesundheitsberichterstattung des Bundes, 2019). All statistical analyses were conducted in SAS statistical software, version 9.4.

## 3 RESULTS

### 3.1 Prevalence of dementia

In 2016, *n* = 213,409 dementia cases were detected by algorithm 1, *n* = 197,400 by algorithm 2, *n* = 48,688 by algorithm 3, *n* = 3033 by algorithm 4, and *n* = 3105 by algorithm 5. This corresponds to age‐ and sex‐standardized prevalence estimates of 3.44 (algorithm 1), 3.19 (algorithm 2), 0.75 (algorithm 3), 0.04 (algorithm 4), and 0.05 (algorithm 5). Table 1 shows the sex‐standardized prevalence rates for any dementia, AD, and VD, stratified by algorithm and age group (the absolute numbers of the persons at risk and the numbers of the prevalent cases are shown in Supplement Table S2). Independently of the dementia subtype, the prevalence rates decreased from algorithm 1–5, with comparable prevalence estimates between AD and VD. Regardless of dementia subtype, each algorithm showed increasing prevalence rates with advancing age. For all subtypes, age‐standardized prevalence estimates were higher in females for algorithms 1 and 2 and comparable to males for algorithms 3–5.

**TABLE 1:** Prevalences (per 100 persons) of Alzheimer's dementia, vascular dementia or any dementia in 2016, stratified by dementia algorithms

Age group (years)	Algorithm 1^a^			Algorithm 2^b^			Algorithm 3^c^			Algorithm 4^d^			Algorithm 5^e^		
	Dementia														
	Any	AD	VD	Any	AD	VD	Any	AD	VD	Any	AD	VD	Any	AD	VD
50–54	0.11	0.02	0.03	0.09	0.01	0.02	0.01	0.00	0.00	0.00	0.00	0.00	0.00	0.00	0.00
55–59	0.21	0.04	0.06	0.18	0.04	0.05	0.03	0.02	0.00	0.01	0.00	0.00	0.01	0.00	0.00
60–64	0.46	0.11	0.14	0.41	0.09	0.12	0.08	0.04	0.01	0.02	0.01	0.00	0.02	0.01	0.00
65–69	0.96	0.24	0.31	0.86	0.21	0.27	0.19	0.10	0.02	0.03	0.01	0.01	0.03	0.01	0.01
70–74	2.51	0.66	0.65	2.27	0.60	0.57	0.65	0.32	0.07	0.07	0.02	0.01	0.07	0.02	0.01
75–79	5.32	1.37	1.24	4.89	1.26	1.13	1.53	0.72	0.17	0.11	0.03	0.02	0.11	0.03	0.02
80–84	11.09	2.75	2.51	10.29	2.50	2.29	3.02	1.33	0.38	0.14	0.04	0.03	0.15	0.04	0.03
85–89	20.12	4.61	4.42	18.86	4.21	4.06	4.26	1.78	0.56	0.11	0.02	0.02	0.12	0.02	0.02
90+	32.10	6.35	6.77	30.37	5.83	6.31	4.15	1.57	0.56	0.06	0.01	0.02	0.06	0.01	0.02
Total^f^	3.44	0.80	0.79	3.19	0.73	0.72	0.75	0.33	0.09	0.04	0.01	0.01	0.05	0.01	0.01
Males^g^	3.20	0.71	0.80	2.96	0.69	0.73	0.77	0.33	0.10	0.05	0.01	0.01	0.05	0.01	0.01
Females^g^	3.52	0.84	0.78	3.27	0.77	0.71	0.73	0.33	0.08	0.04	0.01	0.01	0.04	0.01	0.01

Abbreviations: AD, Alzheimer's dementia; Any, any dementia (including AD; VD, other dementia); VD, vascular dementia.

^a^ At least one inpatient/outpatient diagnosis.

^b^ At least one inpatient diagnosis OR at least one outpatient diagnosis (neurologist) OR two outpatient diagnoses (any specialty).

^c^ Same as (b) with at least one prescription of antidementia drug.

^d^ At least one inpatient/outpatient diagnosis and laboratory testing.

^e^ At least one inpatient/outpatient diagnosis and laboratory testing OR functional imaging.

^f^ Age‐ and sex‐standardized.

^g^ Age‐standardized.

### 3.2 Incidence of dementia

The estimated IRs of dementia per 100 person‐years according to the five algorithms are depicted in Table 2 (the numbers of person‐years and the numbers of incident cases are shown in Suppement. Tables S3 and S4). The overall age‐standardized IRs for any dementia were 1.39 (algorithm 1), 1.13 (algorithm 2), 0.18 (algorithm 3), and 0.05 (algorithms 4 and 5).

**TABLE 2:** Incidences rates (IRs per 100 person‐years) of Alzheimer's dementia, vascular dementia or any dementia in 2015, stratified by dementia algorithms

Age group (years)	Algorithm 1^a^			Algorithm 2^b^			Algorithm 3^c^			Algorithm 4^d^			Algorithm 5^e^		
	Dementia														
	Any	AD	VD	Any	AD	VD	Any	AD	VD	Any	AD	VD	Any	AD	VD
50–54	0.06	0.01	0.01	0.03	0.00	0.01	0.00	0.00	0.00	0.01	0.00	0.00	0.01	0.00	0.00
55–59	0.10	0.02	0.03	0.07	0.01	0.02	0.01	0.00	0.00	0.01	0.00	0.00	0.01	0.00	0.00
60–64	0.20	0.03	0.06	0.14	0.02	0.04	0.02	0.01	0.00	0.02	0.00	0.00	0.02	0.00	0.00
65–69	0.42	0.07	0.12	0.31	0.06	0.09	0.06	0.03	0.01	0.04	0.01	0.01	0.04	0.01	0.01
70–74	1.09	0.18	0.28	0.86	0.15	0.22	0.20	0.09	0.02	0.08	0.02	0.02	0.08	0.02	0.02
75–79	2.18	0.37	0.50	1.78	0.32	0.41	0.42	0.17	0.05	0.12	0.03	0.03	0.12	0.03	0.03
80–84	4.34	0.67	0.98	3.58	0.58	0.82	0.74	0.29	0.09	0.15	0.03	0.04	0.16	0.03	0.04
85–89	7.89	1.07	1.71	6.58	0.92	1.46	0.93	0.34	0.13	0.12	0.03	0.03	0.12	0.03	0.03
90+	12.64	1.59	2.48	10.47	1.33	2.09	0.75	0.26	0.11	0.06	0.02	0.01	0.07	0.02	0.01
Total^f^	1.39	0.21	0.31	1.13	0.17	0.26	0.18	0.07	0.02	0.05	0.01	0.01	0.05	0.01	0.01
Males^g^	1.32	0.19	0.31	1.07	0.16	0.26	0.18	0.07	0.02	0.06	0.01	0.01	0.06	0.01	0.01
Females^g^	1.41	0.21	0.31	1.14	0.18	0.25	0.18	0.08	0.02	0.04	0.01	0.01	0.04	0.01	0.01

Abbreviations: AD, Alzheimer's dementia; Any, any dementia (including AD; VD, other dementia); VD, vascular dementia.

^a^ At least one inpatient/outpatient diagnosis.

^b^ At least one inpatient diagnosis OR at least one outpatient diagnosis (neurologist) OR two outpatient diagnoses (any specialty).

^c^ Same as (b) with at least one prescription of antidementia drug.

^d^ At least one inpatient/outpatient diagnosis and laboratory testing.

^e^ At least one inpatient/outpatient diagnosis and laboratory testing OR functional imaging.

^f^ Age‐ and sex‐standardized.

^g^ Age‐standardized.

Independently of dementia subtype, IRs declined from algorithm 1–5 and increased by advancing age. For all subtypes, age‐standardized IRs were higher in females for algorithms 1 and 2 and comparable to males for algorithms 3–5.

### 3.3 Proportion of dementia subtypes

**FIGURE 2:**
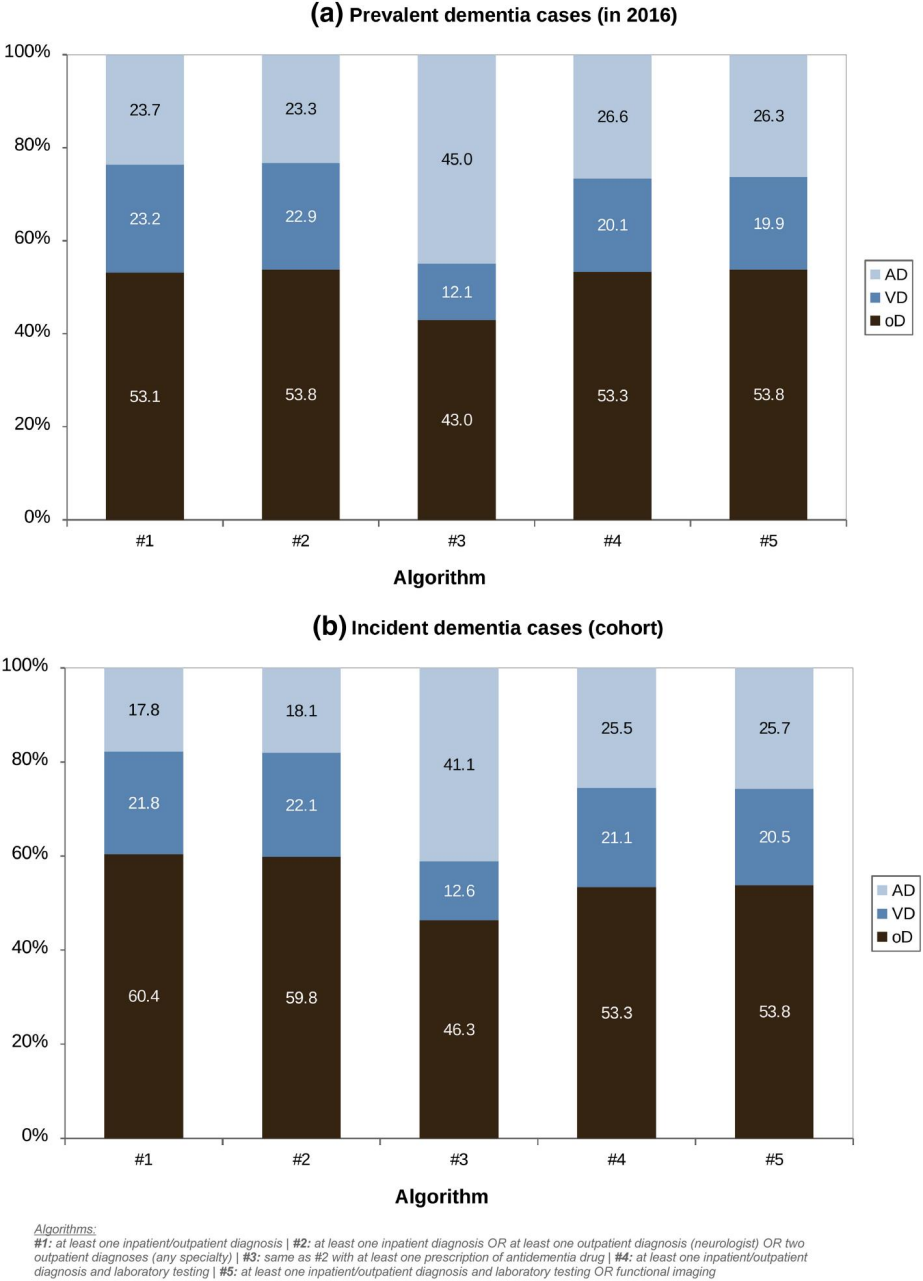
Proportions of dementia subtypes in prevalent (a) and incident (b) dementia cases

The proportions of subtypes among prevalent dementia cases are shown in Figure 2a. With the exception of algorithm 3, all algorithms detected similar proportions of patients with AD (23.3%–26.6%), VD (19.9%–23.2%) or oD (53.1%–53.8%). The proportion of AD patients was higher in algorithm 3 (45.0%) and lower for VD (12.1%) and oD (43.0%).

As with the prevalent dementia cases, there was a similar distribution of the proportions of subtypes in the incident dementia cases (see Figure 2b). For algorithms 1, 2, 4, and 5, similar proportions were found for AD (17.8%–25.8%), VD (20.6%–22.1%), and oD (53.6%–60.4%). For algorithm 3, the proportions were 41.1% for AD, 12.6% for VD, and 46.3% for oD.

## 4 DISCUSSION

Based on data from a large health claims database, we compared prevalence, incidence, and proportions of dementia subtypes according to five algorithms with different levels of strictness. To our knowledge, this study is the first to provide extensive data on the prevalence, incidence, and proportions of dementia subtypes, using a large health claims database of 25 million persons and taking five different algorithms for the detection of dementia into account. Importantly, and following recommendations published by Galeotti et al. (2013), our analyses also considered persons below the age of 65 years, providing insight into the epidemiology of dementia among younger persons.

Common epidemiological patterns could be identified: Regardless of dementia subtype, prevalence and incidence increased by age in the magnitude of doubling every 5 years of life. Due to a decreasing number of cases identified by more restrictive algorithms, we found substantial differences in the prevalence and incidence according to these algorithms. Overall, they were always similar in algorithms 1 and 2 (higher) and 4 and 5 (lower). Prevalence and incidence estimates according to algorithm 3 ranked in between and will be referred to later in the discussion. There were differences in the sex‐specific prevalence estimates which were higher for females according to algorithms 1 and 2, yet comparable to males based on algorithms 3–5. Moreover, with the exception of algorithm 3, each case definition yielded similar prevalence estimates of AD and VD, and algorithms 1 and 2 (and less distinctive algorithms 4 and 5, but not algorithm 3) showed higher incidences of VD than of AD in older patients.

Considering dementia in total (“any”) as identified by algorithms 1 and 2, our estimated prevalence and IRs match well with previously published figures from field studies as well as studies based on health claims data. For example, the regional Leipzig Longitudinal Study of the Aged (LEILA75+) reported slightly lower IRs in those aged 75–79 years than in our data (15.6 vs. 21.8) and slightly higher IRs for the groups beyond 80 years (Riedel‐Heller et al., 2001a). Compared to prevalence estimated in LEILA75+, estimates in our data were higher for those aged 75–79 years (5.3 vs. 3.5%) but similar for higher age groups (Riedel‐Heller et al., 2001b). It is possible that these small differences can be explained by methodological differences such as years under study (1997–1998 vs. 2015–2016) and region of conduct (regional vs. national data). In fact, our findings also coincided with previously reported figures based on analyses with data from Germany's largest statutory health insurance provider, for example, by Stock et al. (2018) who compared the epidemiology of dementia between German and Non‐German insured persons of this provider as well as by Doblhammer et al. who investigated prevalence and incidence between 2007 and 2009 based on the same data source (Doblhammer, Fink, & Fritze 2015; Doblhammer, Fink, Zylla, & Willekens 2015). Compared to results from a Canadian registry‐based study published by Kosteniuk et al. (2015), our prevalence estimates for all‐cause dementia were a little bit higher. This is noteworthy since the authors applied a more broadly algorithm than we have chosen for the GePaRD database (e.g. inclusion of diagnostic codes for amnestic syndromes, unspecified organic or symptomatic mental disorders, omission of treatments with memantine, one single dementia diagnosis was sufficient for inclusion). The incidence estimates cannot be directly compared with our figures due to different units of measurements (persons at risk vs. persony‐years), but the authors reported similar age‐ and sex related patterns for both prevalence and incidence estimates as presented in our study. Their study population can also be considered comparable to ours, regarding other clinical parameters, for example, the proportions of unspecified dementia subtypes or the proportion of patients with inpatient diagnoses (Riedel et al., 2022).

Based upon a study from Catalonia, Ponjoan et al. (2019a) reported lower incidence rates of all‐cause dementia than we have found in GePaRD. In contrast, our prevalence estimates were lower than those reported by Ponjoan et al. (2019a), yet revealed similar age‐ and sex‐related patterns. Overall, our findings are well in line with pooled prevalence estimates from a recently published review and metaanalysis (Bacigalupo et al., 2018). The authors focused on recent studies with a high methodological standard (e.g., use of clinical screening instruments, physician‐based diagnoses), and the fact that no German study could be included in this meta‐analysis underlines the importance of our analyses. However, none of these previously mentioned studies –including those considered by Bacigalupo et al. (2018)– provided estimates for specific dementia entities, and corresponding investigations are still needed. Therefore, in the absence of a basis for comparison, it is difficult to further discuss our specific estimates for AD and VD as presented. In our study, it was an important finding that algorithms 1 and 2 always yielded similar prevalence and incidence estimates, irrespective of dementia subtype. Considering the definition of these algorithms, this implies that the vast majority of dementia cases were either diagnosed in inpatient facilities or by specialized outpatient physicians. Thus, the use of the liberal algorithm one is of similar accuracy as the more restrictive algorithm 2. It should be noted, however, that this does not necessarily hold true for the accuracy in terms of specific dementia diagnoses since these may be subject to change over time (Riedel et al., 2022).

The decrease in case numbers of more restrictive algorithms (leading to corresponding reductions in prevalence/incidence figures) was an interesting secondary result of our analyses. As the cases determined using algorithm 1 represent the maximum possible prevalence of dementia, only about 23% of these cases were treated with antidementia drugs, and supportive diagnostics were documented in the claims data for about 1% of them. The low rate of medication is not particularly surprising and has been reported previously in field studies in the outpatient care sector (Riedel et al., 2012; Riepe & Gaudig, 2010) as well as in studies based on health claims data (Bohlken et al., 2015). They are also plausible in light of the fact that antidementia drugs generally have only very limited efficacy but at the same time, are associated with a relatively broad spectrum of side effects. As a result, physicians potentially refrain from using them more often, at least in advanced stages of the disease (Farlow et al., 2010; Kraft, 2017). However, it should be kept in mind that the treatment situation may vary between countries. For example, a Spanish study recently reported a threefold higher proportion of drug‐treated dementia patients than we have found in our data (Ponjoan et al., 2019b). Several reasons may have accounted for this disparity, such as different national guideline recommendations or different reimbursement policies for medical treatment. Surprising, however, was the low number of patients for whom supportive diagnostics could be identified, since these measures are recommended to confirm/differentiate the diagnosis (Deutsche Gesellschaft für Psychiatrie und Psychotherapie & Deutsche Gesellschaft für Neurologie, 2016; Jessen, 2014). In light of the fact that these assessments are usually more likely to be conducted at disease onset and do not necessarily have to be repeated—thus appearing much less frequently in patients with prevalent dementia—these low numbers may be plausible. However, higher numbers of cases were not found for incident dementia cases either, and the proportions of dementia types, as a possible result of a diagnostic fine differentiation, did not differ from the results of algorithms 1 and 2. Why the use of supportive diagnostics was so rarely found in our data cannot be answered conclusively at this point. International studies have already shown that the vast majority of patients does not receive guideline‐based diagnoses (Nielsen et al., 2011; Phung et al., 2009). This contrasts with the results of a survey study among specialized outpatient physicians, in which 93% of all participants reported regular use of neuroimaging techniques and 27% reported the use of laboratory diagnostics for diagnosing dementia (Lohmann et al., 2017). However, the significance of these results is very limited, as only 8% of the originally contacted specialists participated in the survey, which might point at a substantial response bias. Therefore, in summary, algorithms 4 and 5 do not appear suitable in their current form for detecting dementia patients in health claims data and require further investigation.

Algorithm 3 not only differed from the other algorithms in terms of the epidemiologic estimates as described above, it also yielded nearly twice the proportion of AD and almost half the proportion of VD cases compared to any the other four algorithms. Interestingly, this was true for both prevalent and incident dementia cases, with differences regarding AD being slightly more pronounced in incident than in prevalent patients. This was also reflected by the IRs which were higher for VD than for AD in older patients according to algorithms 1 and 2 but higher for AD according to algorithm 3. This is also supported by findings from a Catalonian study on dementia which was based on health care records (Ponjoan et al., 2019b). The authors compared the validity of AD diagnoses of three different algorithms, using a physician survey as external criterion for diagnostic validation and found a substantial increase of validity, if the patient's treatment status was included. However, other studies (Wilkinson et al., 2018) pointed out that drugs used for dementia nowadays may also be used for other indications. Therefore, the utility of such agents for the identification of dementia cases requires further investigation, to which we hope we could contribute with our study.

However, none of our algorithms yielded dementia proportions as indicated in the Rotterdam study (Ott et al., 1995), neither in prevalent nor in incident dementia cases. In fact, AD accounted for a small proportion of dementias in our data and regardless of which algorithm was applied, oD constituted the biggest fraction among diagnoses, possibly rather due to pragmatic than to clinical reasons (see the discussion of the limitations at the end of this section). Nonetheless, it should be kept in mind that an approximate 70%/20%/10% distribution of AD/VD/oD proportions is by no means a consistent and widely replicated result despite frequent referencing (Prince et al., 2014). Indeed, depending on the setting and methodology, AD proportions comparable to ours or even significantly lower have also been reported. For example, the AD proportion in the German outpatient specialist sector was estimated to be 25%–40% in a survey study (Lohmann et al., 2017). Even lower proportions of around 10% were previously found in health claims data (Albrecht et al., 2018, 2019; Kaduszkiewicz et al., 2014). Cho et al. (2014), who also examined proportions of dementia diagnoses in healthcare data and distinguished between three dementia categories similarly to ours, found 25% AD in incident cases and 31.9% after 1 year of follow‐up. The proportions of VD compared with our results were slightly lower in algorithm 1 (14.9% vs. 23.2%) but comparable in algorithm 3 (14.9% vs. 12.1%). Therefore, against this background, it remains difficult to appraise how closely our results reflect the true distribution of proportions in the population of dementia patients. Nevertheless, our findings suggest that when conducting studies based on health claims data, the inclusion of medication data may potentially improve the detection of AD cases, accepting a reduction in the total number of cases identified. In this context, however, some limitations have to be kept in mind when interpreting our findings.

First and foremost, by using health claims data only, the most serious limitation of our results is the lack of an external criterion, such as matching our dementia cases with “true” dementia cases diagnosed in clinical facilities as gold standard. However, this could not be implemented in our study for several reasons. Measures that could provide further information on a patient's cognitive status (e.g. explicit results from cognitive assessments, screening tests) are not stored in the health claims data, which are mainly collected for billing purposes. To further evaluate our algorithms, we would have needed to link patient data from our health claims database to another data source containing corresponding clinical data, for example, clinical registries. Such registries are not broadly available in Germany to an extent which could adequately cover the dementia cases as detected in our data base. Also, conducting such a linkage directly would not have been possible since all persons included in GePaRD are pseudonymized due to data protection reasons, preventing a retrospective linkage of data from two (or more) data sources for the very same patient. Consequently, our results lack comparative information on the sensitivity and specificity of the individual algorithms, which would be important for assessing their performance. Accordingly, statements about which algorithms “work better” should be treated with great caution, as they can only be based on the results of early studies. Nevertheless, also in light of the lack of data on this to date, we believe that our results can make an important contribution to future studies in which such comparisons are made. This holds especially true in light of a recently published review on 27 epidemiological studies based on data from routine care (Wilkinson et al., 2018). For the large majority of studies considered –which basically applied the same diagnostic ICD‐10 codes like we did in GePaRD– high sensitivities/positive predictive values were reported, with the maximum sensitivity of 86% found when using insurance data (Taylor et al., 2009).

Second, the estimates of dementia were based on patients with at least one diagnostic code of dementia, implying that cases of mild cognitive impairment, which might precede full‐blown dementia, were not considered. This also holds true for undetected dementia cases, which—by definition—do not show up in health claims data, despite possibly representing a substantial proportion among all dementia cases worldwide (Lang et al., 2017). It should also be noted, that nursing home residents with dementia were also included in our study population, since, for example, diagnostic measures performed in the nursing home are also billed as “outpatient” services. However, while previous studies, which were based on registry data and health care records reported an estimated proportion of approximately 25% of demented patients to be nursing home residents (Kosteniuk et al., 2015; Ponjoan et al., 2019a), a reliable deduction about the residential status of the patients was not possible in GePaRD data. Therefore, differentiated analyses (comparison of dementia patients in nursing homes or in a home environment) were not possible for us. Another limitation relates to the fact that GePaRD only contains claims data from individuals with statutory health insurance, whereas individuals with private health insurance are not included in the database. Since private health insurance in Germany is only available for individuals with a high gross annual income or selected occupation groups (e.g. civil servants), insured persons with higher incomes may be underrepresented. However, the figure for privately insured individuals is approximately 11% only, since a large proportion of those who could choose private health insurance due to their income nevertheless voluntarily remain covered by statutory health insurance (Haug & Schink, 2021).

Finally, unlike AD and VD, the category of oD comprised several codes, including “unspecified dementia” which might have accounted for a large proportion of diagnoses in patients with oD. Since AD and VD require extensive (exclusion) diagnostics on the one hand, while on the other hand most dementia diagnoses do not substantially differ in terms of prognosis, treatment or billing conditions for the physician, a rather large proportion of factual AD cases could have been coded as “oD” for mere pragmatic reasons. This limitation is inherent in health claims data and, as indicated above, requires the conduct of further studies that optimize the use of information available in such data by matching them against an appropriate gold standard.

## AUTHOR CONTRIBUTIONS

Oliver Riedel wrote the first draft of the manuscript. Malte Braitmaier conducted all statistical analyses. Malte Braitmaier and Ingo Langner assisted with the interpretation of data. All authors revised the first draft of the manuscript.

## CONFLICT OF INTEREST

Oliver Riedel, Malte Braitmaier, and Ingo Langner are working at an independent, non‐profit research institute, the Leibniz Institute for Prevention Research and Epidemiology—BIPS. Unrelated to this study, BIPS occasionally conducts studies financed by the pharmaceutical industry. Almost exclusively, these are post‐authorization safety studies (PASS) requested by health authorities. The design and conduct of these studies as well as the interpretation and publication are not influenced by the pharmaceutical industry. The study presented was not funded by the pharmaceutical industry and was performed in line with the ENCePP Code of Conduct.

## ETHICS STATEMENT

In Germany, the utilization of health insurance data for scientific research is regulated by the Code of Social Law. All involved health insurance providers as well as the German Federal Office for Social Security and the Senator for Health, Women and Consumer Protection in Bremen as their responsible authorities approved the use of GePaRD data for this study.

## Supporting information

Table S1

Table S2

Table S3

Table S4

## Data Availability

Research data are not shared. As we are not the owners of the data we are not legally entitled to grant access to the data of the German Pharmacoepidemiological Research Database. In accordance with German data protection regulations, access to the data is granted only to employees of the Leibniz Institute for Prevention Research and Epidemiology—BIPS on the BIPS premises and in the context of approved research projects. Third parties may only access the data in cooperation with BIPS and after signing an agreement for guest researchers at BIPS.
