# Supplementary material for: Dementia in health claims data: The influence of different case definitions on incidence and prevalence estimates
Source: Int J Methods Psychiatr Res. 2022 Sep 27;32(2):e1947. doi: 10.1002/mpr.1947 (PMC10242188; doi:10.1002/mpr.1947)
Supplement: Supplementary file 1 — Table S1 [file MPR-32-e1947-s001.docx]

**Supplemental Material**

**Table S1.** Antidementia drugs and supportive diagnostic measurements considered for the dementia case definitions (algorithms 3–5).

| **Antidementia drugs** | Donepezil (ATC-Code: N06DA02) |
| --- | --- |
|  | Rivastigmine (ATC-Code: N06DA03) |
|  | Galantamine (ATC-Code: N06DA04) |
|  | Memantine (ATC-Code: N06DX01) |
| **Supportive diagnostics** | Laboratory tests (OPS-Code:1-204) |
|  | Cranial CT scan (OPS-Codes: 3-200, 3-220) |
|  | Cranial MRI scan (OPS-Codes: 3-800, 3-820) |
|  | Brain PET scan (OPS-Code: 3-740) |
|  | Brain SPECT scan (OPS-Code: 3-720) |
|  | Brain PET/CT scan (OPS-Code: 3-750) |
|  | Brain SPECT/CT scan (OPS-Code: 3-730) |

ATC: Anatomical Therapeutic Chemical; CT: Computer tomography,
MRT: Magnetic resonance imaging, PET: Positron emission tomography,
SPECT: Single photon emission computed tomography
