# Supplementary material for: Dementia in health claims data: The influence of different case definitions on incidence and prevalence estimates
Source: Int J Methods Psychiatr Res. 2022 Sep 27;32(2):e1947. doi: 10.1002/mpr.1947 (PMC10242188; doi:10.1002/mpr.1947)
Supplement: Supplementary file 2 — Table S2 [file MPR-32-e1947-s002.docx]

**Supplemental Table S2.** Numbers of persons at risk, prevalent cases of Alzheimer’s dementia, vascular dementia or any dementia in 2016, stratified by dementia algorithms.

| **Age group (years)** | **Persons**  **at risk** | **Algorithm 1** ^a^ | | |  | **Algorithm 2** ^b^ | | |  | **Algorithm 3** ^c^ | | |  | **Algorithm 4** ^d^ | | |  | **Algorithm 5** ^e^ | | |
| --- | --- | --- | --- | --- | --- | --- | --- | --- | --- | --- | --- | --- | --- | --- | --- | --- | --- | --- | --- | --- |
|  |  | **Any** | **AD** | **VD** |  | **Any** | **AD** | **VD** |  | **Any** | **AD** | **VD** |  | **Any** | **AD** | **VD** |  | **Any** | **AD** | **VD** |
| 50–54 | 1,447,455 | 1,629 | 220 | 374 |  | 1,310 | 181 | 307 |  | 124 | 62 | 12 |  | 56 | 8 | 11 |  | 63 | 11 | 12 |
| 55–59 | 1,255,981 | 2,667 | 559 | 691 |  | 2,268 | 496 | 608 |  | 372 | 236 | 24 |  | 117 | 38 | 14 |  | 125 | 39 | 15 |
| 60–64 | 1,023,396 | 4,746 | 1,119 | 1,462 |  | 4,170 | 970 | 1,279 |  | 790 | 449 | 75 |  | 158 | 52 | 36 |  | 171 | 54 | 38 |
| 65–69 | 886,077 | 8,493 | 2,098 | 2,715 |  | 7,610 | 1,854 | 2,407 |  | 1,689 | 874 | 197 |  | 284 | 81 | 55 |  | 293 | 81 | 56 |
| 70–74 | 741,667 | 18,607 | 4,870 | 4,787 |  | 16,872 | 4,440 | 4,256 |  | 4,798 | 2,393 | 498 |  | 521 | 127 | 106 |  | 543 | 133 | 108 |
| 75–79 | 838,907 | 44,667 | 11,487 | 10,442 |  | 40,987 | 10,530 | 9,454 |  | 12,809 | 6,009 | 1,442 |  | 919 | 254 | 178 |  | 949 | 263 | 181 |
| 80–84 | 452,177 | 50,132 | 12,414 | 11,332 |  | 46,543 | 11,327 | 10,375 |  | 13,668 | 6,017 | 1,702 |  | 651 | 176 | 139 |  | 665 | 177 | 141 |
| 85–89 | 227,303 | 45,723 | 10,485 | 10,057 |  | 42,868 | 9,560 | 9,227 |  | 9,688 | 4,054 | 1,280 |  | 257 | 56 | 52 |  | 264 | 56 | 54 |
| 90+ | 114,477 | 36,745 | 7,265 | 7,753 |  | 34,772 | 6,679 | 7,227 |  | 4,750 | 1,803 | 642 |  | 70 | 14 | 20 |  | 72 | 14 | 21 |
| **Total** | **6,987,440** | **213,409** | **50,517** | **49,613** |  | **197,400** | **46,037** | **45,140** |  | **48,688** | **21,897** | **5,872** |  | **3,033** | **806** | **611** |  | **3,145** | **828** | **626** |
| Males | 3,025,572 | 79,042 | 18,152 | 19,906 |  | 72,971 | 16,452 | 18,179 |  | 20,138 | 8,829 | 2,590 |  | 1,440 | 403 | 294 |  | 1,491 | 404 | 304 |
| Females | 3,961,868 | 134,367 | 32,365 | 29,707 |  | 124,429 | 29,585 | 29,961 |  | 28,550 | 13,068 | 3,282 |  | 1,593 | 403 | 317 |  | 1,654 | 415 | 322 |

^a^ at least one inpatient/outpatient diagnosis

^b^ at least one inpatient diagnosis OR at least one outpatient diagnosis (neurologist) OR two outpatient diagnoses (any specialty)

^c^ same as b) with at least one prescription of antidementia drug

^d^ at least one inpatient/outpatient diagnosis and laboratory testing

^e^ at least one inpatient/outpatient diagnosis and laboratory testing OR functional imaging

Any = any dementia (including AD, VD, other dementia), AD = Alzheimer’s dementia, VD = vascular dementia
