# Supplementary material for: Dementia in health claims data: The influence of different case definitions on incidence and prevalence estimates
Source: Int J Methods Psychiatr Res. 2022 Sep 27;32(2):e1947. doi: 10.1002/mpr.1947 (PMC10242188; doi:10.1002/mpr.1947)
Supplement: Supplementary file 3 — Table S3 [file MPR-32-e1947-s004.docx]

**Supplemental Table S3.** Person-years and numbers of incident cases of Alzheimer’s dementia, vascular dementia or any dementia in 2016, stratified by dementia algorithms.

| **Age group (years)** | **Person-years**^f^ | **Algorithm 1** ^a^ | | |  | **Algorithm 2** ^b^ | | |  | **Algorithm 3** ^c^ | | |  | **Algorithm 4** ^d^ | | |  | **Algorithm 5** ^e^ | | |
| --- | --- | --- | --- | --- | --- | --- | --- | --- | --- | --- | --- | --- | --- | --- | --- | --- | --- | --- | --- | --- |
|  |  | **Any** | **AD** | **VD** |  | **Any** | **AD** | **VD** |  | **Any** | **AD** | **VD** |  | **Any** | **AD** | **VD** |  | **Any** | **AD** | **VD** |
| 50–54 | 1,369,222 | 821 | 96 | 154 |  | 476 | 66 | 96 |  | 42 | 20 | 3 |  | 74 | 15 | 13 |  | 75 | 15 | 13 |
| 55–59 | 1,165,537 | 1,222 | 189 | 299 |  | 787 | 126 | 208 |  | 102 | 52 | 10 |  | 140 | 33 | 30 |  | 150 | 35 | 30 |
| 60–64 | 977,157 | 1,939 | 302 | 568 |  | 1,383 | 229 | 434 |  | 205 | 95 | 20 |  | 169 | 41 | 33 |  | 181 | 43 | 36 |
| 65–69 | 824,612 | 3,440 | 611 | 953 |  | 2,576 | 486 | 742 |  | 512 | 226 | 62 |  | 309 | 67 | 66 |  | 320 | 69 | 66 |
| 70–74 | 775,241 | 8,478 | 1,411 | 2,175 |  | 6,689 | 1,195 | 1,744 |  | 1,525 | 674 | 170 |  | 604 | 153 | 136 |  | 634 | 158 | 140 |
| 75–79 | 748,171 | 16,344 | 2,738 | 3,743 |  | 13,323 | 2,382 | 3,101 |  | 3,183 | 1,298 | 375 |  | 879 | 214 | 194 |  | 909 | 220 | 200 |
| 80–84 | 354,615 | 15,378 | 2,384 | 3,474 |  | 12,762 | 2,059 | 2,926 |  | 2,667 | 1,049 | 334 |  | 542 | 110 | 127 |  | 565 | 119 | 131 |
| 85–89 | 176,107 | 13,893 | 1,881 | 3,019 |  | 11,661 | 1,629 | 2,589 |  | 1,695 | 624 | 237 |  | 217 | 49 | 57 |  | 223 | 53 | 58 |
| 90+ | 73,546 | 9,294 | 1,170 | 1,822 |  | 7,784 | 992 | 1,555 |  | 583 | 202 | 83 |  | 50 | 12 | 9 |  | 51 | 12 | 9 |
| **Total** | **6,464,207** | **70,809** | **10,782** | **16,207** |  | **57,441** | **9,164** | **13,395** |  | **10,514** | **4,240** | **1,294** |  | **2,984** | **694** | **665** |  | **3,108** | **724** | **683** |
| Males | 2,802,344 | 27,525 | 4,097 | 6,731 |  | 22,383 | 3,447 | 5,566 |  | 4,368 | 1,686 | 567 |  | 1,455 | 315 | 352 |  | 1,516 | 327 | 365 |
| Females | 3,661,863 | 43,284 | 6,685 | 9,476 |  | 35,058 | 5,717 | 7,829 |  | 6,146 | 2,554 | 727 |  | 1,529 | 379 | 313 |  | 1,592 | 397 | 318 |

^a^ at least one inpatient/outpatient diagnosis

^b^ at least one inpatient diagnosis OR at least one outpatient diagnosis (neurologist) OR two outpatient diagnoses (any specialty)

^c^ same as b) with at least one prescription of antidementia drug

^d^ at least one inpatient/outpatient diagnosis and laboratory testing

^e^ at least one inpatient/outpatient diagnosis and laboratory testing OR functional imaging

^f^ Since the number of person-years differs slightly between algorithms, the person-years for Algorithm 1 are shown here as an example. For an overview of all person-years (Algorithm 1 to 5) see
 Supplemental Table S4.

Any = any dementia (including AD, VD, other dementia), AD = Alzheimer’s dementia, VD = vascular dementia
