# Supplementary material for: Dementia in health claims data: The influence of different case definitions on incidence and prevalence estimates
Source: Int J Methods Psychiatr Res. 2022 Sep 27;32(2):e1947. doi: 10.1002/mpr.1947 (PMC10242188; doi:10.1002/mpr.1947)
Supplement: Supplementary file 4 — Table S4 [file MPR-32-e1947-s003.docx]

**Supplemental Table S4.** Person-years in the incidence analyses stratified by dementia algorithms.

|  | **Number of person-years (incidence analyses)** | | | | | | | | |
| --- | --- | --- | --- | --- | --- | --- | --- | --- | --- |
| **Age group (years)** | **Algorithm 1** ^a^ |  | **Algorithm 2** ^b^ |  | **Algorithm 3**  ^c^ |  | **Algorithm 4** ^d^ |  | **Algorithm 5** ^e^ |
| 50–54 | 1,369,222 |  | 1,369,406 |  | 1,369,627 |  | 1,369,616 |  | 1,369,616 |
| 55–59 | 1,165,537 |  | 1,165,786 |  | 1,166,135 |  | 1,166,116 |  | 1,166,114 |
| 60–64 | 977,157 |  | 977,448 |  | 978,041 |  | 978,061 |  | 978,057 |
| 65–69 | 824,612 |  | 825,072 |  | 826,119 |  | 826,220 |  | 826,216 |
| 70–74 | 775,241 |  | 776,244 |  | 778,893 |  | 779,323 |  | 779,309 |
| 75–79 | 748,171 |  | 749,833 |  | 755,104 |  | 756,251 |  | 756,237 |
| 80–84 | 354,615 |  | 356,071 |  | 361,269 |  | 362,330 |  | 362,318 |
| 85–89 | 176,107 |  | 177,343 |  | 182,400 |  | 183,147 |  | 183,144 |
| 90+ | 73,546 |  | 74,370 |  | 77,937 |  | 78,199 |  | 78,199 |
| **Total** | **6,464,207** |  | **6,471,572** |  | **6,495,525** |  | **6,499,264** |  | **6,499,209** |
| Males | 2,802,344 |  | 2,805,176 |  | 2,814,290 |  | 2,815,742 |  | 2,815,716 |
| Females | 3,661,863 |  | 3,666,396 |  | 3,681,235 |  | 3,683,521 |  | 3,683,494 |

^a^ at least one inpatient/outpatient diagnosis

^b^ at least one inpatient diagnosis OR at least one outpatient diagnosis (neurologist) OR two outpatient diagnoses (any specialty)

^c^ same as b) with at least one prescription of antidementia drug

^d^ at least one inpatient/outpatient diagnosis and laboratory testing

^e^ at least one inpatient/outpatient diagnosis and laboratory testing OR functional imaging
